# Genetic Analysis of Recombinant Inbred Lines for *Sorghum bicolor × Sorghum propinquum*

**DOI:** 10.1534/g3.112.004499

**Published:** 2013-01-01

**Authors:** Wenqian Kong, Huizhe Jin, Cleve D. Franks, Changsoo Kim, Rajib Bandopadhyay, Mukesh K. Rana, Susan A. Auckland, Valorie H. Goff, Lisa K. Rainville, Gloria B. Burow, Charles Woodfin, John J. Burke, Andrew H. Paterson

**Affiliations:** * Plant Genome Mapping Laboratory, University of Georgia, Athens, Georgia 30602; † USDA-ARS Cropping Systems Research Laboratory, Lubbock, Texas 79401

**Keywords:** quantitative trait locus, simple-sequence repeat, DNA marker, recombination, segregation distortion

## Abstract

We describe a recombinant inbred line (RIL) population of 161 F5 genotypes for the widest euploid cross that can be made to cultivated sorghum (*Sorghum bicolor*) using conventional techniques, *S. bicolor* × *Sorghum propinquum*, that segregates for many traits related to plant architecture, growth and development, reproduction, and life history. The genetic map of the *S. bicolor* × *S. propinquum* RILs contains 141 loci on 10 linkage groups collectively spanning 773.1 cM. Although the genetic map has DNA marker density well-suited to quantitative trait loci mapping and samples most of the genome, our previous observations that sorghum pericentromeric heterochromatin is recalcitrant to recombination is highlighted by the finding that the vast majority of recombination in sorghum is concentrated in small regions of euchromatin that are distal to most chromosomes. The advancement of the RIL population in an environment to which the *S. bicolor* parent was well adapted (indeed bred for) but the *S. propinquum* parent was not largely eliminated an allele for short-day flowering that confounded many other traits, for example, permitting us to map new quantitative trait loci for flowering that previously eluded detection. Additional recombination that has accrued in the development of this RIL population also may have improved resolution of apices of heterozygote excess, accounting for their greater abundance in the F5 than the F2 generation. The *S. bicolor* × *S. propinquum* RIL population offers advantages over early-generation populations that will shed new light on genetic, environmental, and physiological/biochemical factors that regulate plant growth and development.

As a botanical and genomic model for grasses, *Sorghum bicolor* L. Moench. (sorghum), a native of tropical Africa that is the most drought-resistant of the world’s top five cereal crops, is a logical complement to the largely sequenced genome of rice (*Oryza*). Sorghum has biochemical and morphological specializations to improve carbon assimilation at high temperatures (C4 photosynthesis), whereas rice uses C3 photosynthesis more typical of temperate grasses. Like rice, the most recent genome duplication in sorghum appears to be ∼70 million years ago (Paterson *et al.* 2004), simplifying its comparative and functional genomics. With a high-quality DNA sequence (Paterson *et al.* 2009), the ∼740 megabase pair sorghum genome is of high value for better understanding the genome of maize (Schnable *et al.* 2009) and in particular the impact of an ancient tetraploidy in maize shortly after its lineage diverged from that of sorghum (Swigonova *et al.* 2004). Sorghum is of particular importance as a diploid model for the Saccharinae clade of grasses that includes recently formed complex polyploids such as *Saccharum* (sugarcane, currently the world’s no. 1 biofuel crop), and *Miscanthus*, among the greatest-yielding of biomass crops in the U.S. Midwest (Heaton *et al.* 2008). Each of these polyploids share substantial genetic colinearity and synteny with sorghum (Kim *et al.* 2012; Ming *et al.* 1998), and *Saccharum* quantitative trait loci (QTL) often show positional correspondence to those of sorghum (Ming *et al.* 2001, 2002). One of the few crops suited to all proposed approaches for renewable fuel production. *i.e.*, from starch, sugar, and/or cellulose, sorghum itself is presently the no. 2 U.S. source of fuel ethanol from grain (after maize, and is a promising cellulosic biofuel crop (Rooney *et al.* 2007).

*Sorghum bicolor × Sorghum propinquum* is thought to be the widest euploid cross that can be made with the cultigen (*S. bicolor*) by conventional means, and interspecific populations from these species offer opportunities to genetically dissect a wide range of traits related to plant domestication and crop productivity, some of which have begun to receive attention (Chittenden *et al.* 1994; Feltus *et al.* 2006; Hu *et al.* 2003; Lin *et al.* 1995, 1999; Paterson *et al.* 1995a,b). The opportunities offered by comparison of *S. bicolor* and *S. propinquum* have led to much effort to develop genomics resources, including a detailed genetic map (Bowers *et al.* 2003; Chittenden *et al.* 1994), bacterial artificial chromosome-based physical maps for both species (Bowers *et al.* 2005; Draye *et al.* 2001; Lin *et al.* 1999), expressed sequence tag EST) resources (Pratt *et al.* 2005), and a genome sequence (Paterson *et al.* 2009).

Among many other aspects of growth and development, *S. bicolor* and *S. propinquum* differ in characteristics related to perenniality, a life history strategy for which the *Sorghum* genus has become a model (Hu *et al.* 2003; Jang *et al.* 2009; Paterson *et al.* 1995b). Both consideration of how to expand agriculture to provide plant biomass for production of fuels or chemical feedstocks (Tilman *et al.* 2009), and strategies to rebalance food production with preservation of ecological capital (Glover *et al.* 2010), focus heavily on perenniality. Perenniality may also be a curse—*Sorghum halepense*, a wild perennial polyploid resulting from natural hybridization between *S. bicolor* and *S. propinquum*, finds occasional use as forage and even food (seed/flour) but is most noted as one of the world’s most noxious weeds, having spread from its west Asian center of diversity across much of Asia, Africa, Europe, North and South America, and Australia. Demonstration that most genes responsible for variations in size and number in *Sorghum* and *Oryza* of an important perennation organ, the rhizome, map to corresponding chromosomal locations (Hu *et al.* 2003), suggests that information about rhizomatousness from a few models (that are also major crops) may extrapolate broadly to a wide range of taxa.

By single-seed descent from the same *S. bicolor × S. propinquum* F2 population used in early-generation genetic analysis (Lin *et al.* 1995), we have produced and describe here a recombinant inbred line (RIL) population of 161 F5 genotypes that segregate for a wide range of traits, providing a valuable addition to the genetic resources available for this botanical and genomic model. The genetic control of flowering provides an example of how the RIL population contributes to improved knowledge of trait inheritance.

## Materials and Methods

### Genotyping and data analysis

The mapping population comprised 161 F5 RILs derived by selfing of single F2 plants described previously (Lin *et al.* 1995) from a controlled cross between single plants of *S. bicolor* BTx623, and *S. propinquum* (unnamed accession). Leaf samples were frozen at −80° and lyophilized for 48 hr. Genomic DNA was extracted from the lyophilized leaf sample based on Aljanabi *et al.* 1999. Polymerase chain reactions for simple sequence repeat (SSR) analysis were carried out under standard conditions for all primer pairs using 1 U of Taq polymerase with 10× polymerase chain reaction buffer (100 mM Tris-HCl at pH 9, 500 mM KCl, and 15 mM MgCl_2_), 2 mM dNTP, 3 mM MgCl_2_, 0.2 mM of each primer, and 20 ng of DNA template with a final reaction volume of 10 mL. The thermo-cycling was performed with the following program: (1) Preheat at 95° for 3 min, (2) denaturation at 95° for 30 sec, (3) annealing at 65° for 1 min (−1°/ cycle), (4) extension at 72° for 1 min, (5) 10 cycles of steps (2)∼(4), (6) denaturation at 95° for 30 sec, (7) annealing at 55° for 1 min, (8) extension at 72° for 1 min, (9) 32 cycles of steps (6)∼(7), and (10) final extension at 72° for 5 min. The amplified products were visualized in 10% polyacrylamide gels with silver staining.

### Linkage and QTL analysis

A total of 161 F5 individuals were genotyped. MAPMAKER (Lander *et al.* 1987) was used for map construction with the data type ‘ri self,’ which is suitable for the RIL configuration. Heterozygosity in codominant markers was treated as missing data by MAPMAKER because the ‘ri self’ configuration does not recognize it. Map distances, cM, were calculated using the Kosambi function (Kosambi 1944). Marker loci were grouped by two-point linkage analysis with a logarithm of odds ratio (LOD) threshold of 4.0 and a maximum distance of 30 cM. Local maximum likelihood orders of marker loci were confirmed using the ‘ripple’ command. The map was drawn using Adobe Illustrator. In 2009, 2010, and 2011, single 1.5-m plots of each RIL were transplanted (2009, 2011) or direct seeded at the University of Georgia Plant Science Farm, Watkinsville, GA, in a completely randomized design. Flowering dates were recorded for the first five flowers per plot. The average of the first five flowering days was calculated in Microsoft Excel. The means of the flowering dates over years were estimated using best linear unbiased prediction with SAS PROC MIXED. Lines, environmental effect, and their interaction were treated as random. The broad sense heritability (H) was calculated using the variance component method. ($H=\frac{V_{G}}{V_{G}+\frac{V{\;}_{GE}}{e}+\frac{V_{\;residual}}{re}}$). Heritability = 60.822/(60.822 + 102.57/3 + 1.5848/3) = 63.66. QTL analysis used composite interval mapping method in Windows QTL Cartographer V2.5_010 (Wang *et al.* 2012).

Seed of the RIL population are distributed by the U.S. Department of Agriculture-Agricultural Research Service (USDA-ARS), Lubbock, TX (J. Burke).

## Results

### DNA markers and map construction

A total of 203 SSRs initially were selected and scored, derived from sugarcane ESTs (prefix “CA” or “TC”), previously mapped RFLP probe sequences [“Xcup” (Schloss *et al.* 2002)], sorghum-sequenced genomic clones [“Xtxp” (Kong *et al.* 2000)], sorghum EST sequences [“Xisep” (Ramu *et al.* 2009)], previously developed SSRs [“Xgap” (Brown *et al.* 1996)], unpublished SSRs from Agropolis-Cirad-Genoplante (“mSbCIR”), and an unmapped scaffold in the genome sequence. Of those 203 markers, 135 segregating for 141 marker loci were mapped into 10 linkage groups corresponding to the 10 sorghum chromosomes. The remaining markers were excluded due to redundancy (*i.e.*, cosegregation of multiple bands from the same primer) and weak and/or apparent artifactual amplifications. Among the 141 loci mapped in the F5 RILs, there is an average of 9 (5.6%) missing genotypes per locus, with 95% of the loci having less than 29 (18%) missing genotypes. Among 95 loci mapped in the F2 population, there is an average of 25 (6.8%) missing genotypes per locus, with 95% of the loci having less than 103 (27.8%) missing genotypes.

**Figure 1:**
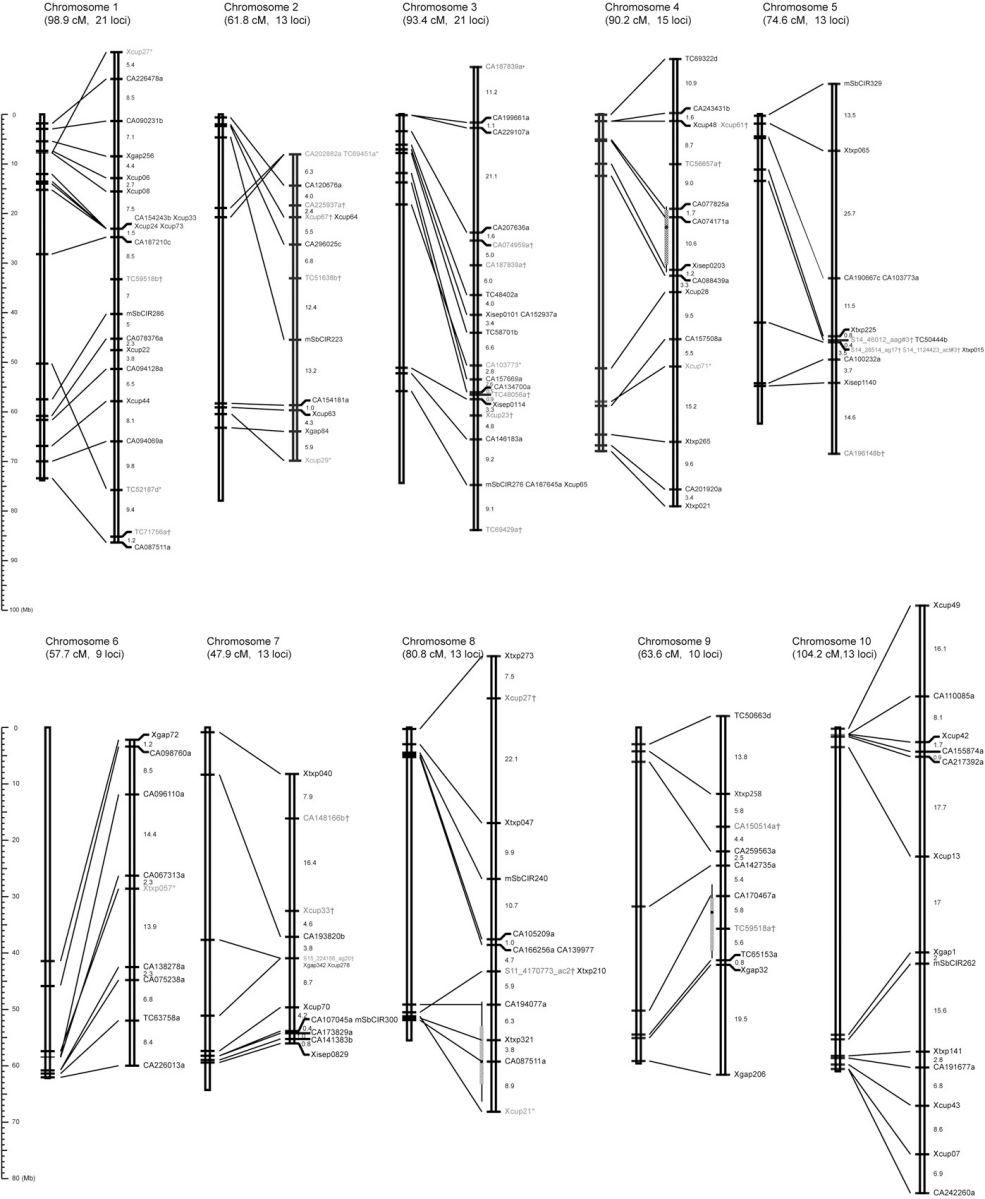
Genetic map of the *S. bicolor* × *S. propinquum* RILs. For each linkage group, genetic distances are shown on the right in Kosambi centimorgans; their corresponding physical chromosomes (from the current genome assembly, as cited) are shown on the left. Markers whose physical positions are unable to be located to their corresponding chromosomes are indicated by †; markers whose genetic orders disagree with their physical positions are indicated by *. Bar and whisker plots on chromosomes 4, 8, and 9 indicate 1- and 2-LOD likelihood intervals for flowering QTL described in the text, with tick marks indicating likelihood peaks.

The genetic map of the RILs derived from annual *S. bicolor* and perennial *S. propinquum* (Figure 1) contains 141 loci on 10 linkage groups collectively spanning 773.1 cM. A total of 35 (24.8%) loci have dominant inheritance, with null alleles from *S. propinquum* at 14 loci and from *S. bicolor* at 21 loci, which is not a significant difference (χ^2^ = 1.4, 1 d.f, *P* = 0.2367). The average interval between consecutive loci is 5.48 cM, ranging from 0.0 cM between cosegregating markers to 25.7 cM in the largest gap (on chromosome 5). Construction of the map used a two-step strategy. First, to minimize ambiguity caused by distorted loci, we constructed a framework map by selecting a subset of clearly scored markers that also did not deviate significantly from the expected Mendelian ratio (1:1) at *P* < 10^−5^ after Bonferroni correction. To assign linkage groups to chromosomes, we anchored framework markers to physical locations by blasting against the sorghum genome sequence. We then assigned and placed additional markers to the framework at LOD score of ≥3.0 and carefully checked for double recombination events in the original scoring data.

### Comparison of genetic map to physical positions

Comparison of the genetic map to physical positions of the mapped loci reveals the relationship between genetic distances and physical distances and the physical distribution of markers along the genome. Each marker on the genetic map was aligned to its corresponding physical position by virtue of the published *S. bicolor* genome sequence [Figure 1 (Paterson *et al.* 2009)]. The sorghum genome sequence information was given priority in ordering markers that were indistinguishable genetically. Overall, a total of 110 of 141 markers in 10 linkage groups are well aligned to their physical positions. The marker order in the genetic map occasionally deviates from that in the physical map. Distal markers on a linkage group tend to disagree more with the physical map than markers in the middle of a group, presumably due to a lack of flanking markers at the ends of chromosomes. A small number of markers show best matches to sequences that are on different chromosomes from where they map genetically. Factors that may contribute to the discrepancies between the genetic and physical maps include multiple amplifications of paralogous loci; sequence assembly errors; or cryptic structural differences between *S. bicolor* and *S. propinquum*.

The mapped SSR marker loci provide substantial coverage of the genetic map, with the exception of chromosome 6 for which markers only cover the lower one third of the chromosome (Figure 1). The unmapped region of this chromosome includes a large heterochromatic block (about 34 Mb) that contains the *S. propinquum Ma1* allele conferring short-day flowering (Lin *et al.* 1995). There is ample polymorphism between the parental genotypes in this region and we mapped the region in the F2 population (Lin *et al.* 1995). However, the RIL population was advanced in a temperate latitude, and artificial selection has largely eliminated photoperiodic flowering. This selection, together with limited recombination in this heterochromatic region, account for it being underpopulated with DNA markers in the RIL map.

Marker distribution is not even along the physical map: markers are concentrated in distal regions and sparse in central regions of the chromosomes. In an extreme case, chromosome 8, a recombinational distance of 4.7 cM spans a physical distance of approximately 46.3 Mb, covering a remarkable 83.4% of the chromosome. This phenomenon is in accordance with our previous observations (Bowers *et al.* 2005; Lin *et al.* 1995; Paterson *et al.* 2009) that the sorghum pericentromeric heterochromatin is recalcitrant to recombination, with the vast majority of recombination occurring in the distal euchromatin.

### Segregation distortion

In the F5 RILs, all chromosomes except chromosome 7 contain regions with segregation distortion significant at the 5% level (Table 1). A total of 14 apices (peak genomic regions) of distortion were found, on chromosome 1 near cM 35.8, chromosome 2 near cM 50.6, chromosome 3 near cM 11.2, 35.0, 66.1, and 84.3; chromosome 4 near cM 77.2, chromosome 5 near cM 0.0 and 60.3; chromosome 6 near cM 0.0; chromosome 8 near cM 39.5; chromosome 9 near cM 26.5 and 37.7; and chromosome 10 near cM 88.7. All regions showed enrichment for *S. bicolor* alleles. Other than the chromosome 6 region under selection for day-neutral flowering, the most striking case of segregation distortion was on chromosome 1—the apex of this distortion was near the locus Xcup24 with a segregation ratio of 154:3 (homozygous *S. bicolor*: *S. propinquum*). This apex was genetically less than 1 cM from the most extreme case found in the F2 population from which these RILs are derived: the locus CSU507 on LG C (Bowers *et al.* 2003). In a larger set of F2 progeny previously described (Lin *et al.* 1995), we found similarly distorted segregation (203:15) in this region.

**Table 1:** Comparison of regions of segregation distortion between *S. bicolor* (SB) × *S. propinquum* (SP) F5 RIL and F2 populations

	F5				F2			
Chr.	Marker	cM	SB:SP	Location, Mb	Marker	SB:SP	LG	Location, Mb
1	Xcup24	35.8	154:3	14.0	pSB195	203:15	C	14.2
2	CA154181a	50.6	129:24	58.3	pSB101	34:74	B	61.6
2	None near				pSB075	111:35	B	66.1
3	CA199661a	11.2	101:40	0.2	None near		A	
3	CA074959a*^a^*	35.0	100:23	3.5- 6.2		N.D.	A	
3	TC48056a*^a^*	66.1	86:43	13.8-51.2		N.D.	A	
3	Xcup65	84.3	85:39	55.9		N.D.	A	
3	None near				pSB443b	128:66	A	69.0
4	Xtxp265	77.2	117:37	64.9	None near		F	
4	N.D.				pSB038	34:101	F	14.2
5	mSbCIR329	0.0	134:26	0.2	None near		H	
5	N.D.				pSB064	33:81	H	6.5
5	Xisep1140	60.3	97:52	54.8		N.D.	H	
6	Xgap72	0.0	151:3	41.4	pSB095	104:60	D	50.7
6	None near				pSB428a	93:41	D	38.0
6	None near				pSB643a	65:20	D	4.3
7	N.D.				pSB784	19:50	J	5.9
8	mSbCIR240	39.5	109:49	4.5		N.D.	E	
9	CA142735a	26.5	115:46	31.7		N.D.	G	
9	TC59518b*^a^*	37.7	105:55	50.2-54.5		N.D.	G	
10	Xcup43	88.7	123:26	59.8	pSB115	124:59	I	60.6

N.D., no distortion (not significantly different from 1:1 segregation); RIL, recombinant inbred line; LG, linkage group.

a Physical location not on the corresponding chromosome of the linkage group: apices are estimated by adjacent marker locations.

We compared the 14 regions of segregation distortion in the F5 RILs to the levels and patterns of segregation found in the F2 population from which these RILs are derived. Because different DNA markers were used in the two studies, this was done by aligning the F2 and F5 genetic maps to their physical locations on the *S. bicolor* genome (Paterson *et al.* 2009). A total of 11 regions of segregation distortion were found in the F2 (Table 1). Four of the 11 regions of segregation distortion in the F2 population favored the *S. propinquum* alleles, among which three are no longer distorted in the F5 RILs, and one region near the end of chromosome 2 contains overrepresentation of *S. bicolor* alleles (!) in the F5 RILs. Those regions with overrepresentation of *S. bicolor* alleles in the F2 generally also contain such overabundance in the F5 RILs, albeit a few cases lack nearby DNA markers. However, eight regions showing normal segregation in the F2 showed overabundance of the *S. bicolor* allele in the F5 RILs.

### Residual heterozygosity

We compared regions of excess/deficiency of residual heterozygosity in the F5 RILs and the F2 population (Table 2). In the F2, eight regions show excess and two show deficiency of heterozygotes. All except two of these also show segregation distortion. In the F5, much higher homozygosity makes it difficult to distinguish heterozygote deficiency with statistical significance but 29 regions show excess, 7 (24%) of which also show segregation distortion. In the F2, the regions showing excess are all small (diagnosed by only 1 marker each); however, a large region of chromosome 1 shows deficiency of heterozygotes. In the F5, there are 3 large regions showing heterozygote excess in chromosomes 4, 5, and 7, respectively.

**Table 2:** Comparison of regions showing over-/underrepresentations of residual heterozygosity between *S. bicolor* × *S. propinquum* F5 RIL and F2 populations

	F5				F2			
Chr.	Marker	cM	H:(SB + SP)*^a^*	Location, Mb	Marker	H:(SB + SP)	LG	Location, Mb
1					pSB102	193:123	C	3.7
1	CA226478a	5.4	21:140	1.8				
1	TC71756a*^b^*	97.7	24:135	47.7/50.3−73.4	SHO68	106:244*^c^*	C	46.8
2	Xcup67*^b^*	12.7	26:133	0.6−2.0				
2	CA296025c	18.2	26:130	2.4				
2	mSbCIR223	37.4	26:131	4.7				
2	Xcup63	51.6	25:136	59.1				
2					pSB101	206:108	B	61.6
2					pSB077	211:103	B	70.0
3	CA152937a	50.2	24:132	7.1				
3	TC48056a*^b^*	66.1	28:129	13.8−51.2				
3	TC69429a*^b^*	93.4	21:135	55.9−end				
3					pSB443b	81:194*^a^*	A	69.0
4	Xcup61*^b^*	12.5	36:120	1.5-5.1				
4	Xisep0203	42.5	26:123	10.0				
5	Xtxp065	13.5	23:131	1.9				
5	CA103773a	39.4	25:135	4.8				
5					pSB064	191:114	H	6.5
5	S14_284514_ag17*^b^*	52.1	41:116	13.5-42.0				
5	CA100232a	55.6	31:130	54.3				
6					pSB643a	178:85	D	4.3
6					pSB140	221:104	D	52.4
6	Xtxp057	26.4	22:134	57.4				
6					pSB487	194:113	D	60.1
7	Xtxp040	0	28:129	0.9				
7					pSB784	127:69	J	5.9
7	Xtxp278	32.7	26:129	51.1				
7	mSbCIR300	45.6	24:137	58.3				
7	Xisep0829	47.8	31:122	59.4				
8	Xtxp047	29.6	31:126	3.0				
8	CA166256a	51.2	23:137	5.3				
8	Xtxp321	68.1	22:134	50.5				
9	TC50663d	0	20:120	3.0				
9	TC65153a	43.3	23:114	54.5				
9	Xgap206	63.6	21:137	59.2				
10	Xcup49	0	30:130	0.2				
10	CA217392a	26.8	21:132	1.6				
10	CA191677a	81.9	21:139	58.6				

RIL, recombinant inbred line; LG, linkage group.

a H:(SB + SP) indicates the ratio of heterozygotes (H) to the sum of parental genotypes (SB + SP).

b Physical location not on the corresponding chromosome of the linkage group: apices are estimated by adjacent marker locations.

c Deficiency of heterozygotes (all other cases are heterozygote excess).

### Initial QTL mapping

**Figure 2:**
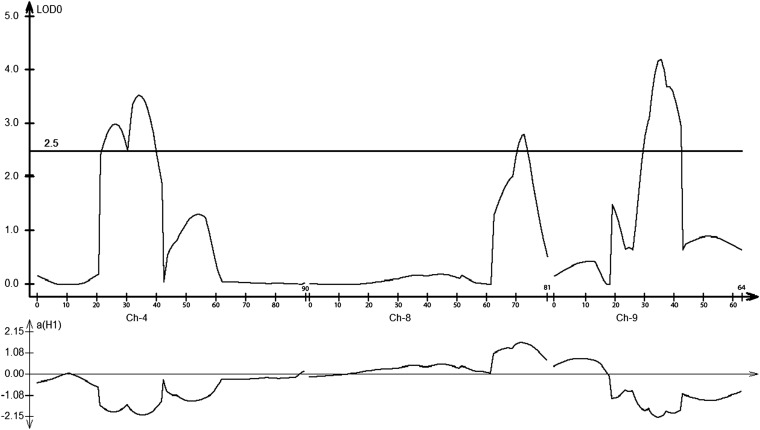
QTL for days to first flower in the *S. bicolor* × *S. propinquum* RILs. Upper plot represents QTL likelihood (LOD scores) at the indicated cM locations on chromosomes 4, 8, and 9. Lower plot indicates additive effect of an allele substitution at the indicated cM locations, calculated based on flowering times associated with *S. bicolor* minus *S. propinquum* alleles (so positive value indicates earliness associated with the late-flowering *S. propinquum* parent).

To explore the merit of the RIL population for QTL mapping, we focused on flowering, a trait associated with the tropical origin of *S. propinquum* that had a large confounding effect on many traits in F2 QTL mapping. In the RIL population, near-homozygosity for the *S. bicolor* allele along the salient portion of chromosome 6 reveals that we have largely eliminated genotypes with short-day flowering alleles from *S. propinquum*. A total of three flowering QTL met a LOD threshold of 2.61 based on 1000 permutation tests on chromosomes 4, 8, and 9 (Figure 2, Table 3). The chromosome 9 QTL found here closely overlaps one found in the F2 generation (Lin *et al.* 1995), which also overlaps a QTL found in several other sorghum populations (Feltus *et al.* 2006; Mace and Jordan 2011). The chromosome 8 QTL also closely corresponds to one found in the BTx623 × IS3620c cross (Brown *et al.* 2006), and the *S. propinquum* allele confers early flowering, accounting for the transgressants we observed in F2 and F5. The chromosome 4 QTL is newly discovered in this population, perhaps “unmasked” as a result of removing short-day flowering but is in a region in which flowering QTL have been reported previously (Mace and Jordan 2011). Indeed, it shows a “double peak” that may indicate the actions of two nearby genes although we presently infer only a single likelihood interval with statistical confidence. Although a previously reported QTL on chromosome 2 (Lin *et al.* 1995) did not reach statistical significance here, there was subthreshold evidence of it (LOD ∼1) in the vicinity that it was previously mapped to.

**Table 3:** Biometric parameters of QTL for days to first flower in the *S. bicolor* x *S. propinquum* RILs

Chr.	LOD	a*^a^*	R2	Start, Mb*^b^*	End, Mb*^b^*	Population/Study (if Not Herein)
2	1	−1.2	0.028	—	—	
2	4.67	−6.7	0.083	61.6	66.1	*S. bicolor × S. propinquum F2* (Lin *et al.* 1995)
4	3.5	−2.01	0.108	5.1	10.0	
8	2.8	1.64	0.072	49.1	51.9	
8	5.5	*^c^*	0.134	50.5	55.5	*S. bicolor BTx623 × IS3620C* (Brown *et al.* 2006)
9	4.2	−2.14	0.114	50.2	54.5	
9	2.53	−10.5	0.042	8.1	57.0	*S. bicolor × S. propinquum F2* (Lin *et al.* 1995)
9	7.7	—	0.195	*^d^*	59.1	*S. bicolor BTx623 x IS3620C* (Feltus *et al.* 2006)

a Additive effect, calculated as *S. bicolor* BTx623 – other (*S. propinquum* or IS3620C as appropriate). To match this system, values reported in (Lin *et al.* 1995) were multiplied by −1.

b Based on flanking DNA marker locations in the published genome sequence (Paterson *et al.* 2009).

c Corresponding values not reported.

d Only a single nearby marker could be definitively mapped to the genome sequence, span of interval uncertain.

## Discussion

The *S. bicolor* × *S. propinquum* RIL population offers advantages over early-generation populations that promises to shed new light on the genetic, environmental, and physiological/biochemical factors that regulate plant growth and development. Dramatic variation in plant architecture, growth and development, reproduction, and life histories of the parental species that segregate among the progeny, together with homozygosity of the RILs and the ability to replicate them across a spectrum of natural and/or controlled conditions, makes this population of high potential importance for the discovery and validation of QTL. Many of these traits have been measured and will be reported under separate cover.

Advancement of the RIL population in a temperate environment (Lubbock, TX) may improve the ability to resolve QTL for traits that were previously below the significance threshold, also providing a more realistic assessment of variation that is relevant to temperate latitudes. For example, near-homozygosity for the *S. bicolor* allele along the salient portion of chromosome 6 reveals that we have largely eliminated genotypes with photoperiod sensitivity (“short-day flowering”), a trait associated with the tropical origin of *S. propinquum* that had a large confounding effect on many traits in F2-based QTL mapping. Eliminating the profound morphophysiological alteration associated with short-day flowering permitted us to identify two flowering QTL that eluded detection in our previous study with 370 F2 plants (Lin *et al.* 1995), one of which accounted for the observation that a few segregants flowered earlier than the early-flowering parent.

The advancement of the RIL population in an environment to which the *S. bicolor* parent was well adapted (indeed bred for) but the *S. propinquum* parent was not may have had some undesirable consequences as well. All segregation distortions in the F5 generation involved excesses of *S. bicolor* alleles, whereas the F2 generation showed similar numbers of cases of *S. bicolor* and *S. propinquum* excess. This finding suggests that in addition to the intended removal of short-day flowering, advancement of the population in temperate continental conditions may have caused some inadvertent selection against other traits of *S. propinquum*, a native of southeast Asia that inhabits streamsides and moist places [(Anonymous 2006) zipcodezoo.com/Plants/S/Sorghum_propinquum/#footref_2]. Although these biases favoring *S. bicolor* alleles may impact the ability to map QTL in a few regions of the genome, the population still exhibits a wide range of morphophysiological variations, with individual lines more comparable to one another by virtue of the near-absence of *Ma1*.

Benefiting from several additional cycles of recombination beyond our previous F2 population, comparison of this genetic map to the sorghum physical map and sequence highlight the striking bias in distribution of recombination across the sorghum genome. This is a good news−bad news scenario—relatively small amounts of physical DNA per cM may facilitate genomic analyses in the gene-rich portions of the genome, but large blocks of recombination-recalcitrant heterochromatin hinder access to other important genes.

Additional recombination that has accrued in the development of this RIL population may have also improved our ability to resolve apices (peak genomic regions) of heterozygote excess, accounting for their greater abundance in F5 than F2 generations, and occurrence in multiple locations on all chromosomes except the one (chromosome 6) for which about two-thirds of the physical length has been fixed due to selection against the *S. propinquum* short-day flowering allele. A remarkably high 29 apices of heterozygote excess, together with rich genetic and genomic tools for these species, may make this an attractive system in which to further dissect the biology underlying interspecific heterozygote advantage.
